# Mr. Charles Bell's Experiments on the Respiratory Nerves

**Published:** 1822-03-01

**Authors:** 


### VIII.

*On the Nerves; giving an Account of some Experiments on their Structure and Functions, which lead to a new Arrangement of the System.* By Charles Bell, Esq. (From the Philosophical Transactions.) Quarto, pp. 30. One Plate. London, 1821.

Without physiology—that is, without a perfect knowledge of the laws by which the *healthy* functions of our system are governed, we cannot expect to make much progress in pathology. Every person, therefore, who elucidates a known, or discovers a new law in the animal economy, contributes his mite to the advancement of the science of medicine—even if that law should not *appear* to bear on any point of pathology or practice at the time.* No man, in this country, works harder in unravelling those mysteries of the nervous system which puzzle our senses, than the present distinguished teacher in the venerable school of the Hunters.

Mr. Bell remarks that, when the physiologist sees two distinct nerves ramifying over the face—three nerves, from different sources, going to the tongue—four to the throat—and nerves in most perplexing intricacy to the neck; when he finds one nerve with numerous ganglia upon it, and another without them—when, in short, after a minute dissection of the nervous system, he finds a mesh, or net-work, spreading every where, it is not surprising that the seeming intricacy and confusion should make him, in despair, resign inquiry. Mr. Bell, however, by long dissection and study, has been able gradually to decipher some of the abstruse language of the nervous system, and hopes, sooner or later, to come to a comprehension of the whole.

The present enquiry is limited to the *nerves of respiration*, which, our author thinks, form a system of great extent, comprehending all the nerves which serve to combine the muscles employed in the act of breathing and speaking. Tranquil breathing gives a very limited view of the respiratory muscles. But if a man be excited by exercise or passion, or by whatever accelerates the pulse, the respiratory action is extended and increased; and, instead of the almost imperceptible motion of the chest, as in common breathing,

* When the air-balloon was first discovered, some one flippantly asked Dr. Franklin what was the use of it? The Doctor answered, in the Socratic manner, by asking another question :—" *What is the use of a new-born infant?—it may become a man.*"

the shoulders are raised at each inspiration, the muscles of the throat and neck are violently drawn, and the lips and nostrils move in time with the general action. If he does not breathe through his mouth, the nostrils expand, and fall in time with the rising and falling of the chest, whilst the curious apparatus of cartilages and muscles of the nose, are as regularly in action as the levator and depressor muscles of the ribs.

" It is quite obvious, that some hundred muscles thus employed in the act of breathing, or in the common actions of coughing, sneezing, speaking, and singing, cannot be associated without cords of connexion or affinity, which combine them in the performance of these actions: the nerves which serve this purpose I call respiratory nerves." P. 6.

Mr. Bell observes that the nerves of all animals, including man, may be divided into two systems or classes—into those destined for the organization necessary to life and motion in an animal—and those which supply organs superadded as the animal advances in the scale of existence. The nerves of the spine, the tenth or sub-occipital nerve, and the fifth or trigeminus, belong to the simple and symmetrical system.

" All these nerves agree in these essential circumstances; they have all double origins; they have all ganglia on one of their roots; they go out laterally to certain divisions of the body; they do not interfere to unite the divisions of the frame; they are all muscular nerves, ordering the voluntary motions of the frame; they are all exquisitely sensible; and the source of the common sensibility of the surfaces of the body: when accurately represented on paper, they are seen to pervade every part; no part is without them; and yet they are symmetrical and simple as the nerves of the lower animals.

" If the nerves be exposed in a living animal, those of this class exhibit the highest degree of sensibility; while, on the contrary, nerves not of this original class or system, are comparatively so little sensible, as to be immediately distinguished; in so much that the quiescence of the animal suggests a doubt whether they be sensible in any degree whatever. If the *fifth nerve and the portio dura of the seventh*, be both exposed on the face of a living animal, there will not remain the slightest doubt in the mind of the experimenter which of these nerves bestows sensibility. If the nerve of this original class be divided, the skin and common substance is deprived of sensibility; but if a nerve not of this class be divided, it in no measure deprives the parts of their sensibility to external impression." P. 10.

The nerves connecting the internal organs of respiration with the sensibilities of remote parts, and with the respiratory muscles are distinguished from the above by many circumstances. They have not double roots, nor ganglia on their origins. They come off from the *medulla oblongata* and the upper part of the spinal marrow, and from this origin they diverge to those several remote parts of the frame which are combined in the motion of respiration. These are the nerves which give the appearance of confusion to the dissection, because they cross the others, and go to parts already plentifully supplied from the other system. The following are *respiratory nerves*, according to their functions.

1. The *par vagum*, distributed to the larynx, lungs, heart, and stomach, associating these organs together, though they are plentifully supplied with nerves from other sources.

"Comparative anatomy would lead us to infer that this nerve is not essential to the stomach, as it does not exist but where there are heart and lungs to associate with a muscular apparatus of respiration. That the stomach must be associated with the muscular apparatus of repiration, as well as the lungs, is obvious, from the consideration of what takes place in vomiting and hiccough, which are actions of the respiratory muscles excited by irritation of the stomach." P. 11.

2. *Respiratory Nerve of the Face*, or portio dura of the seventh. This nerve also goes off from the *medulla oblongata*, spreads wide on the face, and on it *solely* depend all those motions of the nostril, lips, and face generally (as will presently be shewn) which accord with the motions of the chest in respiration. By the division of this nerve the face is deprived of its consent with the lungs, and of all expression of motion.

3. *Superior Respiratory Nerve* of the trunk, or spinal accessory, which has puzzled physiologists on account of the singular course which it pursues. After arising from the upper part of the spinal marrow, in a line with the roots of the other respiratory nerves, it passes into the skull, and comes out with the par vagum, descending upon the neck, supplying muscles of the shoulder already profusely supplied with nerves from other sources. This nerve controls the operations of the muscles of the neck and shoulder in their office as respiratory muscles, when by lifting the shoulders they take the load from the chest, and give freedom to the expansion of the thorax. When this nerve is cut across in experiments, the muscles of the shoulder, which are in action as respiratory muscles, cease their co-operation, but remain capable of voluntary actions.

4. *The internal Respiratory Nerve*—phrenic or diaphragmatic. This is the only nerve of the system usually considered respiratory. Its origin, course, and destination are familiar to all. But there is another nerve much resembling it which has been entirely overlooked. It is—

5. *The external Respiratory Nerve*, which has its origin with the preceding nerve, coming from the cervical vertebræ, and being connected with the phrenic nerve. It runs down the neck, crosses the cervical and axillary nerves, passes through the axilla, and arrives on the outside of the ribs, where, it is hardly necessary to observe, the muscles are already supplied by nerves coming out betwixt the ribs from the system of regular nerves.

"These four last mentioned nerves govern the muscles of the face, neck, shoulders, and chest, in the actions of excited respiration, and are absolutely necessary to speech and expression. But there are other nerves of the same class which go to the tongue, throat, and windpipe, no less essential to complete the act of respiration. These are the glosso pharyngeal nerve, the lingual, or ninth of Willis, and the branches of the par vagum to the superior and inferior larynx." P. 13.

The nerves of the face afford the best illustration of the foregoing doctrines. The human countenance performs many functions—mastication, breathing, natural voice and speech, expression of the passions and emotions.

*Trigeminus.* In all animals that have a stomach, with palpi or tentacula to embrace their food, the rudiments of this nerve are to be observed. From the nerve that comes off from the anterior ganglion of the leech, and which supplies its mouth, we may trace up through the gradations of animals a nerve of taste and manducation, until we arrive at the complete distribution of the fifth or trigeminus in men. It comes off from the base of the brain, in so peculiar a situation as to receive roots from the medullary process of the cerebrum and cerebellum. It has a ganglion near its origin. This nerve, as will be shewn, serves for taste, motion, and common sensibility in the tongue, jaws, and face.

*Portio Dura of the 7th Pair.* This nerve does not exist, except where there is some consent of motions established betwixt the face and the respiratory organs. It arises close to the nodus cerebri, in a line with the roots of the other respiratory nerves. While within the temporal bone two cords of communication are formed with the branches of the 5th nerve—the vidian and corda tympani. By these communications nerves go in both directions—branches of the seventh are sent to the membrane of the nose, and to the muscles at the back of the palate—while branches of the fifth nerve, and also of the sympathetic, are brought into the interior of the ear. By the corda tympani, branches of this respiratory nerve have access to the velum palati and its muscles. After the nerve emerges on the face every anatomist knows what an extensive range it takes, penetrating to all the muscles—muscles already amply supplied by branches of the fifth pair. The descending or inferior branches of the portio dura going under the lower jaw and to the superficial muscles of the throat and neck, are connected with branches of the spinal nerves, and with the respiratory nerves.

The *structure* of this nerve Mr. Bell has found to differ from that of the fifth pair, and to correspond with that of the par vagum.

The question now naturally occurs, whether these nerves perform the same function? whether they furnish a double supply of the *same* property or endowment—or whether they do not perform different offices? This question is now to be decided by experiment.

" *Experiment.* An ass being thrown, and its nostrils confined for a few seconds, so as to make it pant and forcibly dilate the nostrils at each inspiration, the portio dura was divided on one side of the head; the motion of the nostril of the same side instantly ceased, while the other nostril continued to expand and contract in unison with the motions of the chest.

" On the division of the nerve, the animal gave no sign of pain; there was no struggle nor effort made when it was cut across.

" The animal being untied, and corn and hay given to him, he eat without the slightest impediment.

" An ass being tied and thrown, the superior maxillary branch of the fifth nerve was exposed. Touching this nerve gave acute pain. It was divided, but no change took place in the motion of the nostril; the cartilages continued to expand regularly in time with the other parts which combine in the act of respiration; but the side of the lip was observed to hang low, and it was dragged to the other side. The same branch of the fifth was divided on the opposite side, and the animal let loose. He could no longer pick up his corn; the power of elevating and projecting the lip, as in gathering food, was lost. To open the lips the animal pressed the mouth against the ground, and at length licked the oats from the ground with his tongue. The loss of motion of the lips in eating was so obvious, that it was thought a useless cruelty to cut the other branches of the fifth.

" This experiment of cutting the respiratory nerve of the face, or *portio dura*, gave so little pain, that it was several times repeated on the ass and dog, and uniformly with the same effect. The side of the face remained at rest and placid, during the highest excitement of the other parts of the respiratory organs.

"When the ass, on which the respiratory nerve of the face had been cut, was killed, which was done by bleeding, an unexpected opportunity was offered of ascertaining its influence, by the negation of its powers on the side of the face where it was cut across." 18.

In this ass, where the respiratory facial nerve was cut, the most remarkable contrast was exhibited in the two sides of the face, while the animal was dying; for, whilst the one side was in universal and powerful contraction, the other, where the nerve was divided, remained quite placid.

"From these facts we are entitled to conclude, that the *portio dura* of the seventh, is the respiratory nerve of the face; that the motions of the lips, the nostrils, and the velum palati are governed by its influence, when the muscles of these parts are in associated action with the other organs of respiration. These passages to the lungs are membranous tubes, moved by muscles, which serve to expand and widen them, so that the air may freely enter into the lungs. It is obvious that to produce this, these muscles must have a consent with the other muscles of respiration, and move simultaneously with them; and this is effected through the respiratory nerve of the face. It shall be proved in the sequel, that the throat, neck, shoulders, and chest, have similar nerves to this, similar in structure and function, and that these unite all the extended apparatus of breathing and speaking." 19.

The actions of sneezing and coughing are entirely confined to the influence of the respiratory nerves. When carbonate of ammonia was put to the nostrils of the ass, that side of the nose and face where the nerves were entire, was curled up with the peculiar expression of sneezing; but, on the other side, where the nerve was divided, the face remained quite relaxed, although the branches of the fifth pair and sympathetic were entire. The same effect was produced in a dog.

"These last experiments show, that the peculiar expression in sneezing, results from an impression on the respiratory nerves, and that the muscles of the face are drawn into sympathy solely by the influence of the respiratory nerve of the face." 20.

Mr. Bell cut the respiratory nerve on one side of the face of a monkey, and the peculiar activity of his features on that side, ceased altogether. The timid motions of his eyelids and eyebrows were lost, and he could not wink on that side. His lips were drawn to the other side, like a paralytic drunkard, whenever he shewed his teeth in rage.

"We have proofs," says Mr. Bell, "equal to experiments, that in the human face the actions of the muscles which produce smiling and laughing, are a consequence of the influence of this respiratory nerve. A man had the trunk of the respiratory nerve of the face injured by a suppuration, which took place anterior to the ear, and through which the nerve passed in its course to the face. It was observed that, in smiling and laughing, his mouth was drawn in a very remarkable manner to the opposite side. The attempt to whistle was attended with a ludicrous distortion of the lips; when he took snuff and sneezed, the side where the suppuration had affected the nerve remained placid, while the opposite side exhibited the usual distortion." 21.

Thus it appears that, whenever the action of any of the muscles of the face is associated with the act of breathing, it is performed through the operation of this nerve. Mr. Bell cut a tumour from before the ear of a coachman, and divided a branch of the nerve which goes to the angle of the mouth. Sometime afterwards the man returned to thank Mr. Bell for ridding him of a formidable disease, but complained that he could no longer whistle to his horses.

Mr. Bell makes many ingenious observations and experiments on the functions of the trigeminus or fifth pair, to which we cannot do justice in this place.

"By an experiment made on the 16th of March, it was found, that on cutting the infra-orbitary branch of the fifth nerve on the left side, and the *portio dura*, or respiratory, on the right side of an ass, the sensibility to pain on the right side, where the *portio dura* of the seventh nerve was cut, remained entire, while that of the left side was completely destroyed by the division of the fifth. It was also apparent in this experiment, as in the others, that there was the most marked difference in the sufferings of the animal, when these nerves were cut across. The cutting of the fifth nerve gave pain in a degree corresponding with our notions of the sensibility of nerves; but in cutting the *portio dura*, it was not evident that the animal suffered pain at all." 22.

Some people may ask, to what does this discussion lead after all?—It may be answered, that both the surgeon and physician are interested in knowing that two sets of nerves are distributed to the face, having distinct functions. To the surgeon, this knowledge must prove useful, in performing operations on the face, as well as in observing the symptoms of disease; but to the physician these facts are peculiarly important, as—

"He will be better able to distinguish between that paralysis which proceeds from the brain, and that partial affection of the muscles of the face, when, from a less alarming cause, they have lost the controlling influence of the respiratory nerve.

"Cases of this partial paralysis must be familiar to every medical observer. It is very frequent for young people to have what is vulgarly called a blight; by which is meant, a slight palsy of the muscles on one side of the face, and which the physician knows is not formidable. Inflammations of glands seated behind the angle of the jaw will sometimes produce this. All such affections of the respiratory nerve will now be more easily detected; the patient has a command over the muscles of the face, he can close the lips, and the features are duly balanced; but the slightest smile is immediately attended with distortion, and in laughing and crying the paralysis becomes quite distinct." 25.

The knowledge of the sources of expression, teaches us to be more minute observers. There are conditions of the lungs, Mr. Bell remarks, where the patient is in great danger, and yet the inflammation is not marked by the usual signs of pain and difficult motion of the chest. We shall see nothing but the twitching of those muscles of the face which are animated by the respiratory nerve. We see a certain unusual dilatation of the nostrils, and a constrained motion of the lips, which, with the change of voice, are just sufficient to give alarm, and indicate the patient's condition. "This is a state of the lungs very often produced after severe accidents, as gun-shot wounds, and great surgical operations."

Mr. Bell hopes, soon to lay before the Royal Society, an account of the nerves of the throat, neck, and chest, and to be able to distinguish and separate the nerves of respiration, amid the apparent intricacy of the general system.

"By cutting across these nerves of respiration, we shall find it possible successively to stop the motions of the several parts, which unite in the act of respiration; not only to stop the motion of the diaphragm, but the motions of the side, of the shoulder, of the larynx or the pharynx, by cutting their respective respiratory nerves. When this is done, they will be left in the exercise of their other functions through their other nerves, and still alive to other excitements, and capable of performing the voluntary motions, though dead to the influence of the heart and lungs." 27.

An excellent engraving of the nerves of the face accompanies this paper. We shall not fail to take early notice of Mr. Bell's succeeding communications, and have no doubt of his throwing considerable light on one of the most difficult and intricate subjects of physiological anatomy.

